# Effects of dupilumab on nasal mucociliary clearance in severe CRSwNP: evaluation of the saccharin test as an objective outcome measure

**DOI:** 10.1007/s00405-026-10606-0

**Published:** 2026-09-29

**Authors:** Giulio Pagliuca, Veronica Clemenzi, Salvatore Martellucci, Edoardo Covelli, Andrea Stolfa, Vanessa Di Stefano, Andrea Gallo, Valentina Terenzi

**Affiliations:** 1 Otolaryngology University Unit - “S. M. Goretti Hospital”, Latina, Italy; 2 Otolaryngology University Unit - “Riuniti di Anzio-Nettuno”, Anzio, Italy; 3 Department of Neuroscience, Mental Health and Sensory Organs, Faculty of Medicine and Psychology, “Sapienza” University of Rome, Rome, Italy; 4 Department of Surgery, Maxillo- facial Surgery Unit, University of Rome Tor Vergata, Rome, Italy; 5 Via Mario Fascetti 5, Rome, 00136 Italy

**Keywords:** Nasal polyps, Rhinosinusitis, Monoclonal antibodies, Mucociliary clearance, Rhinitis, Dupilumab, Saccharin test

## Abstract

**Objective:**

Chronic rhinosinusitis with nasal polyps (CRSwNP) is a chronic, relapsing condition associated with a significant impairment in quality of life. Persistent inflammation contributes to dysfunction of mucociliary clearance. Dupilumab (IL-4Rα inhibitor) is a well-established therapeutic agent that has demonstrated significant efficacy in reducing type 2 inflammation and improving quality of life in patients with severe CRSwNP refractory to medical and/or surgical treatment.

This study aimed to evaluate whether dupilumab therapy improves mucociliary clearance in patients with CRSwNP.

**Methods:**

Twenty-one patients with severe CRSwNP were enrolled. All received dupilumab (300 mg subcutaneously every 2 weeks) according to prescribing criteria. At baseline and after 4, 8, and 12 weeks, patients underwent evaluation with SNOT-22, nasal endoscopy with Nasal Polyp Score (NPS), and assessment of mucociliary clearance using the saccharin test (ST). Nasal mucociliary clearance time (NMCT) values were compared across time points..

**Results:**

Significant improvements were observed in SNOT-22, NPS, and NMCT. Mean NMCT values measured using the ST values decreased from 14.3 minutes at baseline to 10.1 minutes at 4 weeks (p<.01), 7.75 minutes at 8 weeks, and 5.84 minutes at 12 weeks, indicating a progressive and statistically significant enhancement of mucociliary clearance.

**Conclusions:**

Short-term dupilumab therapy was associated with a significant improvement in nasal mucociliary clearance in patients with severe CRSwNP. These preliminary findings support the potential role of the saccharin test as a simple, objective tool for monitoring response to biologic therapy.

## Introduction

CRSwNP remains a challenging condition for ENT specialists due to the complexity of managing the underlying chronic inflammatory process that characterizes the disease [1, 2]. Treatment protocols with biological drugs (monoclonal antibodies targeting IL-4, IL-5, and IgE signaling) are widely used to act on the immunological mechanisms underlying the disease [3, 4] in patients with severe uncontrolled CRSwNP.

Dupilumab is a fully human monoclonal antibody that targets the alpha subunit of the IL-4 receptor (IL-4Rα, type I and type II) and inhibits IL-4 and IL-13 signaling. Previous studies have shown that dual inhibition of IL-4 and IL-13 represents an effective therapeutic strategy for CRSwNP, demonstrating significant efficacy of dupilumab in improving the main signs and symptoms of the disease [5].

A significant reduction in mucociliary clearance in patients with nasal polyposis has been widely reported in the literature [6]. Mucociliary clearance is an innate defense mechanism of the respiratory mucosa in which ciliated and goblet cells trap and transport environmental pathogens toward the throat, where they are swallowed or expectorated. In healthy individuals, NMCT does not normally exceed 20 min, while impaired NMCT has been highlighted in rhinitis, sinusitis, asthma and cystic fibrosis [7, 8]. Studies have highlighted that impaired ciliary motility is associated with epithelial hyperplasia observed in individuals with nasal polyps. This impairment is thought to result from persistent type 2 mucosal inflammation commonly found in patients with CRSwNP [9].

We hypothesize that reducing inflammation in patients with CRSwNP treated with dupilumab may lead to an improvement in mucociliary clearance. This improvement could be quantified using the saccharin test (ST). The ST is a non-invasive and reproducible method used to evaluate mucociliary clearance by measuring the time required for a sweet-tasting particle (saccharin) to reach the pharynx and be perceived by the patient after being placed on the inferior turbinate [10–12].

Currently, the clinical response to dupilumab therapy in patients with CRSwNP is typically evaluated using the Nasal Polyp Score (NPS) or subjective measures such as the SNOT-22 and smell tests [13]. The aim of this study was to investigate whether treatment with dupilumab can improve nasal mucociliary clearance time (NMCT) and to explore the potential role of the saccharine test as a monitoring measure of mucociliary transport in patients with CRSwNP undergoing dupilumab therapy.

## Materials and methods

### Study design and population

Twenty-one patients with severe CRSwNP were prospectively enrolled between May and October 2022 at the “A. Fiorini” General Hospital (Terracina) and the “Santa Maria Goretti” General Hospital (Latina), Sapienza University of Rome, Italy. At baseline, demographic data and detailed medical history focusing on disease-specific clinical features were collected prior to treatment initiation (Table 1). The study was approved by the local Ethics Committee (Comitato Etico Lazio 2; protocol no. 0082270/2022).

**Table 1:** General characteristics of the study population

	Overall (*n*: 21)
Age, yr	55 (44–66)
Sex	
M	13 (61.9%)
F	8 (38.1%)
NSAID intolerance	4 (19.0%)
Asthma	18 (85.7%)
No of previous surgeries	
1	11 (52.4%)
2	7 (33.3%)
≥ 3	3 (14.3%)

## Eligibility criteria

Inclusion criteria were: age ≥ 18 years; non-smoker status; diagnosis of CRSwNP confirmed by nasal endoscopy; severe disease (Nasal Polyp Score [NPS] ≥ 5 and/or SNOT-22 ≥ 50); inadequate symptom control with intranasal corticosteroids; and failure of prior medical therapy (≥ 2 courses of systemic corticosteroids in the previous year due to inefficacy or intolerance) and/or previous endoscopic sinus surgery (ESS).

Exclusion criteria included: age < 18 years; smoking or use of medications potentially affecting nasal mucociliary clearance time (NMCT) (e.g., analgesics, sedatives, nasal decongestants); radiotherapy or chemotherapy within 12 months prior to enrollment; and long-term systemic corticosteroid therapy for chronic autoimmune diseases.

## Treatment protocol and clinical assessment

All patients received dupilumab (300 mg subcutaneously every 2 weeks) according to approved prescribing criteria. Clinical evaluation was performed at baseline and after 4, 8, and 12 weeks of treatment (Time 0, 1, 2, 3 respectively). At each time point, patients completed the Sino-Nassal Outcome Test questionnaire (SNOT-22), underwent nasal endoscopy for NPS assessment, and mucociliary clearance evaluation.

## Saccharin test (ST)

Nasal mucociliary clearance time was assessed using the saccharin test (ST). The procedure was standardized, and an identical protocol was used for all assessments. All measurements were performed by one of two experienced examiners (VC or AS) during the morning. The saccharin test was carried out in the same outpatient examination room, with patients evaluated in the same sitting position throughout the follow-up. The procedure was initiated only after patients had reached a comfortable position. Assessments were performed under similar environmental conditions in the same air-conditioned outpatient examination room.

Patients were seated comfortably with the head slightly extended. The more patent nasal cavity (consistently used for all subsequent measurements) was illuminated using a headlight and a nasal speculum. A saccharin particle (~ 1 mm sodium saccharin) was placed on the medial surface of the inferior turbinate, approximately 1 cm posterior to its anterior end, using bayonet forceps. Timing commenced immediately after placement. Patients were instructed to report the first perception of a sweet taste. During the test, subjects were asked to refrain from sniffing, sneezing, eating, drinking, coughing, or blowing their nose. Tests were interrupted and rescheduled if these conditions were not met or if no perception occurred within 60 min.

### Statistical analysis

Statistical analyses were performed using Numiqo (Numiqo, Austria). Continuous variables are presented as mean ± standard deviation (SD). Normality was assessed using the Shapiro-Wilk test. Changes in NPS, NMCT, and SNOT-22 scores across the four evaluation time points (baseline, 4, 8, and 12 weeks) were assessed using a one-factor repeated-measures analysis of variance (ANOVA). The assumption of sphericity was assessed using Mauchly’s test. When sphericity was violated, the Greenhouse-Geisser correction was applied to adjust the degrees of freedom. When the overall ANOVA was statistically significant, pairwise comparisons between time points were performed using the Bonferroni post hoc correction for multiple testing. Effect sizes were estimated using partial eta squared (η²p). As a sensitivity analysis, the non-parametric Friedman test was also performed. Changes from baseline to week 12 were calculated as the baseline value minus the week-12 value; therefore, higher positive change scores indicated greater improvement, including greater mucociliary transport efficiency for NMCT. Associations among changes in NMCT, SNOT-22, and NPS were assessed using Spearman’s rank correlation coefficient. The Holm correction was applied to account for the three pairwise correlation analyses.

No prospective sample-size or power calculation was performed. All statistical tests were two-tailed, and a p value < 0.05 was considered statistically significant.

## Results

Table 2 summarizes all parameters collected at each scheduled ENT evaluation. SNOT-22 scores, NPS and ST were compared at baseline and after 4, 8, and 12 weeks (time 1, time 2, and time 3, respectively). Assessment of normality using the Shapiro-Wilk test showed that NMCT values were normally distributed at all evaluation time points (all *p* > 0.31). Slight deviations from normality were observed for SNOT-22 at baseline (W = 0.835, *p* = 0.002) and for NPS at weeks 8 (W = 0.836, *p* = 0.002) and 12 (W = 0.901, *p* = 0.037). No significant departures from normality were detected for SNOT-22 at weeks 4, 8, and 12 or for NPS at baseline and week 4 (all *p* > 0.05).

**Table 2:** Results from a repeated-measures ANOVA (Analysis of Variance) across the four time periods are statistically significant at the conventional alpha levels of 0.05. This indicates significant differences between the means of the four time points for all analyzed parameters

Outcome	ε	df1	df2	F	*p*	η^2^*p*
SNOT-22	0.605	1.82	36.31	187.43	< 0.001	0.90
NPS	0.548	1.64	32.86	111.64	< 0.001	0.85
ST	0.584	1.75	35.05	156.06	< 0.001	0.89

*SNOT-22* sino-nasal outcome test, *NPS* nasal polyp score, *ST* saccharin test, ε greenhouse_geisser epsilon, *df1* numerator degrees of freedom, *df2* denominator degrees of freedom, *η*^2^*p* partial eta squared

Mauchly’s test indicated that the assumption of sphericity was violated for SNOT-22 (W = 0.329, χ²(5) = 20.36, *p* = 0.001), NPS (W = 0.097, χ²(5) = 42.82, *p* < 0.001), and ST/NMCT (W = 0.270, χ²(5) = 23.98, *p* < 0.001). Therefore, Greenhouse–Geisser corrections were applied to all three repeated-measures ANOVAs. As a sensitivity analysis, the non-parametric Friedman test confirmed significant changes over time for SNOT-22 (χ² = 63.00, *p* < 0.001), NPS (χ² = 55.13, *p* < 0.001), and NMCT (χ² = 63.00, *p* < 0.001), supporting the robustness of the repeated-measures ANOVA results.

After Greenhouse-Geisser correction, repeated-measures ANOVA confirmed a significant effect of time on SNOT-22 (ε = 0.605; F(1.82, 36.31) = 187.43, *p* < 0.001, η²*p* = 0.904), NPS (ε = 0.548; F(1.64, 32.86) = 111.64, *p* < 0.001, η²*p* = 0.848), and ST/NMCT (ε = 0.584; F(1.75, 35.05) = 156.06, *p* < 0.001, η²*p* = 0.886).

**Fig. 1:**
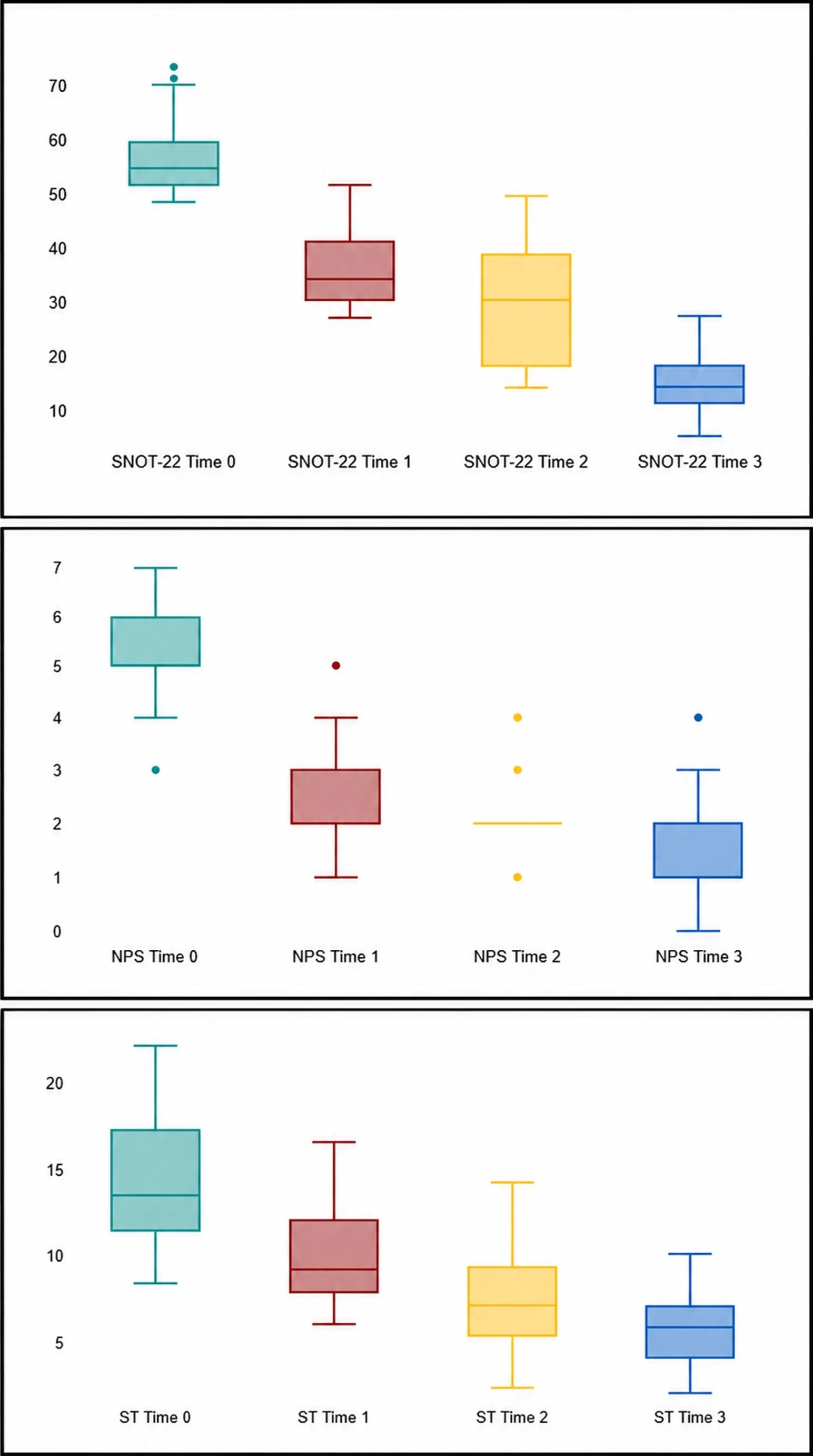
Boxplots illustrating the distribution of SNOT-22, Nasal Polyp Score (NPS), and Saccharin Test (ST) values at baseline (Time 0) and during follow-up (times 1–3). Median SNOT-22, NPS and ST scores progressively decreased from baseline to Time 3, reflecting an overall improvement in patient-reported symptoms, endoscopic disease severity and nasal mucociliary clearance

Mean SNOT-22 scores improved from 57.19 ± 7.72 to 14.57 ± 6.10 (F = 187.43, *p* < 0.001, η²*p* = 0.90) while NPS scores progressively decreased from 5.24 ± 1.04 at baseline to 1.67 ± 1.20 at the final follow-up (F = 111.64, *p* < 0.001, η²*p* = 0.85). Similarly, ST time significantly decreased from 14.28 ± 4.00 to 5.84 ± 2.11 (F = 156.06, *p* < 0.001, η²*p* = 0.89) (Fig. 1). Post hoc Bonferroni analysis demonstrated significant differences between all pairwise comparisons across the four time points (all *p* ≤ 0.034 for NPS; all *p* ≤ 0.001 for ST time and SNOT-22), indicating a progressive improvement throughout the follow-up (Table 3). Large effect sizes were observed for all three outcome measures, with time accounting for 85–90% of the observed variance.

**Table 3:** Bonferroni post-hoc-tests. All pairwise comparisons between time points are statistically significant after Bonferroni correction. The mean difference increases progressively from Time 0 to Time 3

		Mean diff.	Std. Error	t	*p*	95% CI lower limit	95% CI upper limit
SNOT-22							
Time 0	Time 1	20.57	1.98	10.40	< 0.001	16.45	24.70
Time 0	Time 2	27.14	2.28	11.91	< 0.001	22.39	31.90
Time 0	Time 3	42.62	1.06	40.08	< 0.001	40.40	44.84
Time 1	Time 2	6.57	1.46	4.51	0.001	3.53	9.61
Time 1	Time 3	22.05	1.66	13.32	< 0.001	18.59	25.50
Time 2	Time 3	15.48	2.21	7.01	< 0.001	10.87	20.08
NPS							
Time 0	Time 1	2.43	0.20	12.02	< 0.001	2.01	2.85
Time 0	Time 2	3.14	0.14	22.00	< 0.001	2.84	3.44
Time 0	Time 3	3.57	0.23	15.21	< 0.001	3.08	4.06
Time 1	Time 2	0.71	0.23	3.10	0.034	0.23	1.19
Time 1	Time 3	1.14	0.30	3.77	0.007	0.51	1.77
Time 2	Time 3	0.43	0.11	3.87	0.006	0.20	0.66
ST							
Time 0	Time 1	4.18	0.48	8.75	< 0.001	3.18	5.18
Time 0	Time 2	6.54	0.54	12.21	< 0.001	5.42	7.65
Time 0	Time 3	8.44	0.53	15.82	< 0.001	7.33	9.55
Time 1	Time 2	2.35	0.19	12.19	< 0.001	1.95	2.76
Time 1	Time 3	4.26	0.29	14.77	< 0.001	3.66	4.86
Time 2	Time 3	1.90	0.31	6.11	< 0.001	1.25	2.55

*SNOT-22* sino-nasal outcome test, *NPS* nasal polyp score, *ST* saccharin test

Time 0: baseline; Time 1: after 4 weeks; Time 2: after 8 weeks; Time 3: after 12 weeks

No treatment-related adverse events were reported during the study period. At the individual-patient level, improvement in NMCT was inversely correlated with improvement in SNOT-22 (ρ = −0.635, Holm-adjusted *p* = 0.006) and NPS (ρ = −0.467, Holm-adjusted *p* = 0.033). Conversely, improvements in SNOT-22 and NPS were positively correlated (ρ = 0.532, Holm-adjusted *p* = 0.026).

## Discussion

dupilumab is the first biologic approved in both the EU and the USA for the treatment of patients with severe CRSwNP inadequately controlled by standard therapies. Its mechanism of action involves inhibition of IL-4 and IL-13 signaling, leading to a downstream reduction of key type 2 inflammatory mediators [14].

Previous studies demonstrated that dupilumab, as an add-on to intranasal corticosteroids, provides early and sustained clinically meaningful improvements in quality of life, while reducing the need for systemic corticosteroids and sinonasal surgery compared with placebo [14, 15]. In the phase III SINUS-24 and SINUS-52 trials, dupilumab treatment resulted in significant improvements across multiple disease domains, including nasal polyp size, sinus opacification, and key symptoms such as nasal obstruction, discharge, and loss of smell. Notably, these benefits were maintained up to 52 weeks, highlighting the importance of sustained suppression of type 2 inflammation for long-term disease control.

Our findings are in line with the existing literature, confirming a significant, rapid and progressive reduction in both Nasal Polyp Score (NPS) and SNOT-22 scores, already evident at 4 weeks after treatment initiation and further improved over time. The early decrease in NPS observed in our cohort reinforces the clinical effectiveness of dupilumab in reducing polyp burden, while the parallel improvement in patient-reported outcomes reflects a meaningful impact on disease-related quality of life.

In the present study, we applied the ST—to the best of our knowledge, not previously investigated in this context—to evaluate changes in nasal mucociliary clearance efficiency in patients with severe CRSwNP undergoing dupilumab therapy. Our aim was to explore the potential role of this simple and objective test in the follow-up of these patients.

Mucociliary clearance time assessed by the saccharin test has been reported in healthy subjects to range approximately between 7 and 15 min, although values up to 20 min may still be considered within broad physiological limits depending on the population and methodological variability [6–8, 16, 17]. In this context, baseline NMCT values in our cohort (14.3 min) were at the upper limit of the normal range, suggesting borderline impaired mucociliary function in patients with severe CRSwNP. The progressive reduction observed during dupilumab therapy, reaching 5.8 min at 12 weeks, indicates an improvement in mucociliary transport efficiency, even beyond average values reported in healthy controls. These findings are difficult to explain, and further experiences are warranted to better elucidate this trend. It would also be of interest to investigate whether the relatively low mucociliary clearance values observed in our cohort were influenced by ethnic or geographical factors. All our patients were recruited from a relatively homogeneous geographical area that is predominantly rural, distant from major urban centers, and characterized by a climate moderated by its proximity to the Mediterranean Sea.

nevertheless, our results support the concept that biologic therapy may improve epithelial function and enhance mucociliary clearance in patients with severe type 2 inflammatory disease. A significant reduction in NMCT was observed as early as 4 weeks after treatment initiation, indicating a rapid improvement in mucociliary function. This effect may be attributable to the suppression of type 2 inflammation mediated by biological therapy. These findings support the potential utility of the saccharin test as a practical and objective tool for routine monitoring of treatment response in patients receiving dupilumab. Although departures from normality were observed for SNOT-22 at baseline and for NPS at weeks 8 and 12, the consistency between the Greenhouse-Geisser-corrected repeated-measures ANOVA and the non-parametric Friedman test supports the robustness of the observed treatment effects.

Although NMCT, SNOT-22, and NPS all improved significantly at the group level, Spearman’s rank correlation analyses with Holm-adjusted p values showed that changes in NMCT did not parallel symptomatic or endoscopic improvements at the individual-patient level. These findings are quite unexpected and difficult to explain. The limited 12-week follow-up may have conditioned our results. Studies with longer follow-up and repeated assessments are needed to determine whether changes in NMCT, SNOT-22, and NPS become more closely aligned over time. The observed correlations may also have been influenced by the small sample size, the test-retest variability of the saccharin test, the subjective nature of the SNOT-22, and the restricted range of NPS. Therefore, these results should be considered exploratory and do not establish the convergent validity of the saccharin test. Larger studies specifically designed to assess its validity and responsiveness are required.

Majima et al. evaluated NMCT in both healthy subjects and patients with CRSwNP using the saccharin test (ST), demonstrating significantly prolonged clearance times in the latter group [16]. Similarly, Passali et al. reported that inferior turbinate hypertrophy and septal deviation do not significantly affect NMCT, whereas markedly delayed clearance was observed in patients with chronic sinusitis [17]. They hypothesized that chronic inflammation of the paranasal sinuses increases mucus viscoelasticity through the release of inflammatory mediators, along with a reduction in the periciliary layer, ultimately impairing coordinated ciliary activity.

Dysregulated mucociliary clearance and increased mucus production are now recognized as key contributors to the pathogenesis of CRS [17]. In this context, pendrin—an epithelial anion transporter involved in mucus hypersecretion—plays a relevant role [18]. Increased expression of pendrin has been demonstrated in nasal polyps of patients with CRSwNP and is associated with impaired mucociliary clearance. Notably, pendrin expression is upregulated by type 2 cytokines, particularly IL-4 and IL-13, which are central to CRSwNP pathophysiology and may promote inflammation, mucus overproduction, and reduced mucociliary function [19].

In our study, the saccharin test showed a significant and progressive reduction in NMCT, suggesting that biologic therapy improves mucociliary clearance. This effect may be explained by the inhibition of IL-4 and IL-13 signaling mediated by dupilumab, which likely attenuates pendrin overexpression and its downstream effects on mucus production and ciliary function. Although pendrin expression was not assessed in this study, future prospective studies with larger cohorts should combine serial assessment of mucociliary clearance with analysis of pendrin expression in nasal tissue. Correlating pendrin levels with NMCT, endoscopic findings, and symptom scores may help establish whether pendrin represents a mechanistic biomarker of impaired mucociliary function and a potential predictor of response to dupilumab.

All patients included in the study maintained stable intranasal corticosteroid therapy throughout the 12-week follow-up, in accordance with current clinical practice, and no major treatment modifications were recorded. No patient received systemic or topical antibiotic therapy or systemic corticosteroids during follow-up. Concomitant saline irrigation was not standardized as part of the study protocol; therefore, its potential confounding effect cannot be completely excluded. Biologic therapy in combination with topical corticosteroids is currently considered the standard of care for patients with CRSwNP who have failed surgical and/or maximal medical treatment [3–5]. Given the broad consensus supporting this therapeutic approach, together with its well-established efficacy and safety profile, the inclusion of an untreated or alternatively treated control group was neither feasible nor ethically justifiable especially for a real-life study. Consequently, our analysis was limited to the longitudinal evaluation of outcomes within the treated cohort, precluding direct comparisons with a control group. The absence of an untreated or comparator group limits causal inference and is an important limitation of this study. Alternative explanations for the observed improvement as regression to the mean, spontaneous temporal variability in nasal mucociliary clearance, placebo, or expectation effects, cannot be completely excluded. Randomized controlled studies specifically evaluating mucociliary clearance outcomes would be required to confirm causality.

Another limitation of the present study is the small sample size. No prospective sample-size or power calculation was performed because of the exploratory nature of the study and the limited number of eligible patients treated during the enrollment period. Given the small sample size and the evaluation of multiple outcomes across several time points, the possibility of both type I and type II errors cannot be excluded, and the findings should therefore be interpreted with caution. However, larger real-world cohorts are expected to become available in the near future as the use of biologic therapy continues to expand.

All assessments were conducted in the same air-conditioned examination room, using a consistent patient position and the same nasal side throughout follow-up; however, temperature and humidity were not formally recorded or strictly matched between visits. Although all assessment were performed during the morning, the exact time of testing was not formally recorded. Moreover, measurements were performed by one of two experienced examiners, who were not blinded to the visit sequence or previous test results.

Test–retest reliability was not directly assessed in the present study because repeated measurements before treatment initiation were not available. Although the saccharin test was performed under standardized protocol, repeated measurements with ST may have been influenced by increasing patient and examiner familiarity with the procedure. Therefore, although a reduction of NMCT due to the learning effect associated with repeated testing cannot be completely excluded, the results observed in our cohort (approximately 60% from baseline to week 12) exceeded the variability reported for the saccharin test [7], making it unlikely that the observed improvement was solely attributable to repeated testing.

Finally, our investigation was restricted to the assessment of mucociliary clearance in patients receiving dupilumab. Future studies should also evaluate the effects of other currently approved biologic agents to determine whether the observed findings are consistent across different therapeutic mechanisms.

Several authors have proposed practical monitoring protocols to assess patient adherence and quantify symptom improvement during biologic therapy [20]. However, to the best of our knowledge, the saccharin test (ST) has not yet been systematically evaluated as a tool for objectively monitoring response to dupilumab. ST is a simple, safe, rapid, well-tolerated, and reproducible method for assessing mucociliary clearance. Although further validation is required, it may represent a potentially useful adjunct in future monitoring protocols for patients undergoing biologic therapy, providing an additional objective measure alongside clinical and patient-reported outcomes.

## Conclusions

All patients evaluated over the 12-week follow-up showed improvement in saccharin test (ST) results. These findings suggest that dupilumab may contribute to an improvement in nasal mucociliary clearance in appropriately selected patients with CRSwNP and type 2 inflammation.

However, given the limited sample size and short follow-up, these results should be interpreted with caution. Further studies, ideally involving larger cohorts and randomized controlled designs, are warranted to confirm these observations and to better define the role of the saccharin test as a safe, reproducible, and objective tool for monitoring response to biologic therapy in patients with nasal polyposis.
